# Systemic inflammation enhances stimulant-induced striatal dopamine elevation in tobacco smokers

**DOI:** 10.1016/j.bbi.2022.08.016

**Published:** 2022-09-01

**Authors:** Yasmin Zakiniaeiz, Jocelyn Hoye, Joseph Ryan Petrulli, Brittany LeVasseur, Gelsina Stanley, Hong Gao, Soheila Najafzadeh, Jim Ropchan, Nabeel Nabulsi, Yiyun Huang, Ming-Kai Chen, David Matuskey, Daniel S. Barron, Benjamin Kelmendi, Robert K. Fulbright, Michelle Hampson, Kelly P. Cosgrove, Evan D. Morris

**Affiliations:** a Department of Psychiatry, Yale School of Medicine, New Haven, CT, USA; b Yale Positron Emission Tomography (PET) Center, Yale School of Medicine, New Haven, CT, USA; c Department of Radiology and Biomedical Imaging, Yale School of Medicine, New Haven, CT, USA; d Department of Psychiatry, Brigham & Women’s Hospital, Boston, MA, USA; e Department of Anesthesiology, Perioperative, and Pain Medicine, Brigham & Women’s Hospital, Boston, MA, USA; f Department of Biomedical Engineering, Yale School of Medicine, New Haven, CT, USA

**Keywords:** Lipopolysaccharide, Endotoxin, Cigarette smoking, Dopamine elevation, Positron emission tomography

## Abstract

Immune-brain interactions influence the pathophysiology of addiction. Lipopolysaccharide (LPS)-induced systemic inflammation produces effects on reward-related brain regions and the dopamine system. We previously showed that LPS amplifies dopamine elevation induced by methylphenidate (MP), compared to placebo (PBO), in eight healthy controls. However, the effects of LPS on the dopamine system of tobacco smokers have not been explored. The goal of Study 1 was to replicate previous findings in an independent cohort of tobacco smokers. The goal of Study 2 was to combine tobacco smokers with the aforementioned eight healthy controls to examine the effect of LPS on dopamine elevation in a heterogenous sample for power and effect size determination. Eight smokers were each scanned with [^11^C]raclopride positron emission tomography three times—at baseline, after administration of LPS (0.8 ng/kg, intravenously) and MP (40 mg, orally), and after administration of PBO and MP, in a double-blind, randomized order. Dopamine elevation was quantified as change in [^11^C]raclopride binding potential (ΔBP_ND_) from baseline. A repeated-measures ANOVA was conducted to compare LPS and PBO conditions. Smokers and healthy controls were well-matched for demographics, drug dosing, and scanning parameters. In Study 1, MP-induced striatal dopamine elevation was significantly higher following LPS than PBO (*p* = 0.025, 18 ± 2.9 % vs 13 ± 2.7 %) for smokers. In Study 2, MP-induced striatal dopamine elevation was also significantly higher under LPS than under PBO (*p <* 0.001, 18 ± 1.6 % vs 11 ± 1.5 %) in the combined sample. Smoking status did not interact with the effect of condition. This is the first study to translate the phenomenon of amplified dopamine elevation after experimental activation of the immune system to an addicted sample which may have implications for drug reinforcement, seeking, and treatment.

## 1. Introduction

Animal, genetic, postmortem, and imaging data suggest that immune-brain interactions influence the pathophysiology of a broad range of neurologic and psychiatric diseases (Heneka et al., 2015), including epilepsy (Vezzani and Granata, 2005), depression (Hodes et al., 2015; Byrne et al., 2016), and most relevant, addiction (Crews et al., 2017; Kelley and Dantzer, 2011). Studies have demonstrated that drugs and alcohol interact with the neuroimmune system and alter neuroimmune gene expression and signaling (Frank et al., 2011; Haroon et al., 2012), which in turn contribute to various reinforcing and addictive properties of addiction including reward, stress, habit formation, and decision-making (Koob and Volkow, 2010). Further, many immune molecules interact with neurotransmitter systems and play essential roles in modulating synaptic function. For example, inflammatory cytokines, such as interferon-alpha (IFN-α), and their signaling pathways regulate the synthesis, packaging, release, and/or reuptake of several neurotransmitters, including dopamine (Heinisch and Kirby, 2010; Rostène et al., 2007). Understanding the mechanisms by which immune activation causes and contributes to addiction could have a broad impact on improving treatment strategies, including identifying impediments to successful treatment and/or identifying new pharmacological targets such as immunomodulating drugs.

It is well-established that experimental manipulation of systemic inflammation produces effects on the human brain, especially areas involved in reward (i.e., striatum) (Eisenberger et al., 2010; Harrison et al., 2015), and the dopamine system (Dunn, 1992; Felger et al., 2013; Petrulli et al., 2017; Felger, 2018). Immune activation can be reliably elicited with lipopolysaccharide (LPS), also called endotoxin, an agonist of the toll-like receptor 4 (TLR4). LPS-induced activation of TLR4 causes systemic inflammation (Hannestad et al., 2012), activation of microglia (Sandiego et al., 2015), and changes in neuronal activity (Eisenberger et al., 2010) and behavior (Woodcock et al., 2021; Ghahremani et al., 2012; Robertson et al., 2015). LPS is also titratable, making it an excellent model that can be given in a precise and repeatable dose (per bodyweight) and delivered consistently across subjects and groups.

LPS serves as a biological stressor causing elevated blood pressure, heart rate, cortisol, cytokines, and negative emotion (Hannestad et al., 2012; Sandiego et al., 2015; DellaGioia et al., 2013; Hannestad et al., 2012; Sorrells and Sapolsky, 2007). These symptoms caused by LPS are also symptoms experienced due to psychosocial stress and other stressors. Stress is known to increase dopamine transmission in striatum (Petrulli et al., 2017; Imperato et al., 1989; Wand et al., 2007) and the likelihood of smoking relapse (McKee et al., 2011; Sinha, 2001). An elevated dopamine response in the striatum is linked to important behavioral characteristics of tobacco smoking, such as inhibitory control (Colzato et al., 2007; Monterosso et al., 2005) and impulsivity (Bari and Robbins, 2013). Therefore, LPS is a relevant model for stress-induced smoking relapse. Findings from animal studies have linked LPS to drug reinstatement via three main findings: (1) LPS administration causes elevation of the major stress hormone, corticosterone, in rats, (2) reinstatement of cocaine-seeking occurs following corticosterone administration in rats trained to self-administer cocaine, and (3) corticosterone plus cocaine administration enhances dopamine transmission in ventral striatum compared to cocaine alone using microdialysis. However, much less is known about the molecular events of these effects in humans and specifically, how dopamine mediates the effects of systemic inflammation in tobacco smokers.

Previous animal and human studies that examined the effects of inflammation on dopamine did not use the well-established LPS model, but rather, used a pre-treatment with inflammatory cytokines. Rhesus monkeys pre-treated with the cytokine IFN-α, an inflammatory stimulus that increases peripheral cytokines and acts as a biological stressor, showed higher dopamine levels after acute (2 weeks) and lower dopamine levels after chronic (4 weeks) treatment relative to saline, using microdialysis (Felger et al., 2013). PET imaging of rhesus monkeys treated with 4 weeks of IFN-α showed decreased dopamine release relative to saline in the putamen (Felger et al., 2013) which can be restored by levodopa (Felger et al., 2015). In another study, individuals with the hepatitis C virus treated with IFN-α for 4–6 weeks exhibited significantly increased [^18^F]DOPA uptake and decreased [^18^F]DOPA turnover in reward-related regions, including caudate, putamen, and ventral striatum (Capuron et al., 2012). Functional magnetic resonance imaging (fMRI) of the same subjects under the same protocol showed reduced ventral striatal activation during a hedonic reward task (Capuron et al., 2012). These findings suggest that dopamine neuro-transmission in reward-related regions such as the striatum is altered by acute and chronic inflammation.

Studies that did use the LPS model to examine effects on dopamine were limited to animals, non-molecular methods such as fMRI, and healthy control individuals. In rodents, acute systemic immune activation with LPS produced increases in dopamine release (Dunn, 1992). fMRI imaging of healthy control subjects showed that ventral striatal activity in response to reward was reduced by an acute dose of LPS (0.8 ng/kg, 2–3 h prior to scanning) (Eisenberger et al., 2010). Our group was the first to show that LPS, compared to placebo (PBO), amplified methylphenidate (MP)-induced dopamine transmission in striatum in healthy control subjects using PET (Petrulli et al., 2017). These findings suggest that reward activity may be altered following acute LPS-induced immune activation. Based on the ability of LPS to act as a biological stressor and studies showing that stress increases dopamine transmission in striatum (Petrulli et al., 2017; Imperato et al., 1989; Wand et al., 2007) and the likelihood of smoking relapse (McKee et al., 2011; Sinha, 2001), it is important to translate these findings to tobacco smokers.

We previously demonstrated the ability of the LPS model to induce acute systemic inflammation (Petrulli et al., 2017) in healthy control subjects. To determine the wider validity of the LPS model, we sought to replicate and translate this finding in an independent sample of tobacco smokers. In the present study, we examined the effects of LPS administration on MP-induced dopamine elevation in two related studies. In Study 1, we measured the effects of LPS administration on MP-induced dopamine elevation (MP + LPS) relative to PBO (MP + PBO) in tobacco smokers to replicate and translate previous findings from healthy control individuals as part of an ongoing study. In Study 2, after determining that our groups were well-matched on demographics, drug dosing, and scanning parameters, we combined our data in smokers with our previously published data in healthy control individuals (Petrulli et al., 2017), for power and effect size determination in a heterogenous sample. Based on our previous study (Petrulli et al., 2017) and the ability of the LPS model to act as a biological stressor, we hypothesized that LPS administration would amplify MP-induced dopamine elevation in *a priori* (striatum) and exploratory (caudate, putamen, and ventral striatum) regions of interest (ROIs). To our knowledge, this is the first study to examine the phenomenon of amplified dopamine elevation after experimental activation of the immune system in an addicted sample which may have implications for drug reinforcement, seeking, and treatment (McKee et al., 2011; Sinha, 2001).

## 2. Materials and methods

This study was approved by Yale University’s Human Investigation Committee, Radioactive Drug Research Committee, and Radiation Safety Committee. Informed consent was obtained prior to the performance of any study procedures.

### 2.1. Study 1:

#### 2.1.1. Subjects

Nine individuals who smoke tobacco (referred to as smokers) (6 female) aged 32 ± 3.6 years (mean ± SE) were studied. Subjects had no history or evidence of significant medical disorders on physical exam and did not meet DSM-5 criteria for current or past psychiatric or substance abuse diagnosis (except nicotine dependence). Smokers smoked 15 ± 2.9 cigarettes per day for 16 ± 4.2 years and had Fagerström Test for Nicotine Dependence (FTND) (Etter et al., 1999) scores of 5.8 ± 0.8, indicating moderate dependence (Table 1). On intake day, smoking status was confirmed by spirometry to measure carbon monoxide (CO) levels > 11 parts per million (ppm) and by urine samples to measure cotinine—the primary metabolite of nicotine—levels > 150 ng/ml (NicAlert cotinine test strips; Nymox Pharmaceutical). On scan day, overnight abstinence was confirmed by CO levels < 10 ppm or ≤ 50 % of their intake level. One male smoker was excluded due to overnight sleep disturbance on the MP + PBO scan day which has been shown to impact dopamine receptor availability (Volkow et al., 2012) (See Supplemental Material; Table S1). Pregnancy and lactation were exclusionary.

#### 2.1.2. Study design

**Fig. 1.**
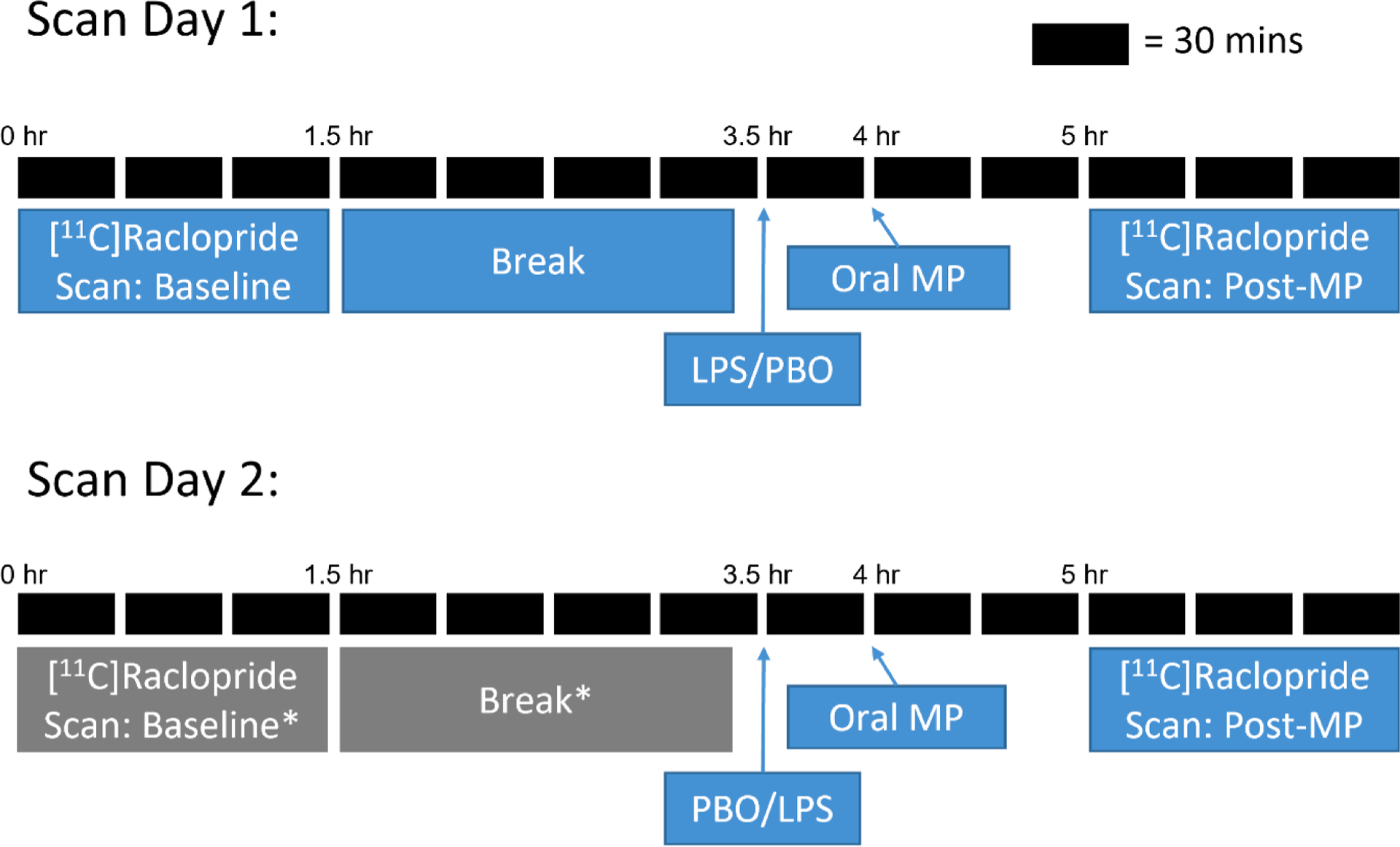
Study Design: The blue boxes refer to the protocol for all the smokers in Study 1. Grey (marked with asterisks (*) and blue boxes combined, represent the protocol for healthy controls, included in Study 2.

Each subject underwent a structural MRI scan on a Prisma 3T MR scanner (Siemens/CTI, Knoxville, TN, USA) for anatomical localization. Subjects also participated in two PET scan sessions on two separate days (Fig. 1). Each scan session consisted of either a pair of PET scans: a baseline scan and an MP + LPS/PBO scan; or just an MP + LPS/PBO scan. The baseline scan was a 90-min [^11^C]raclopride scan. MP + LPS/ PBO scans consisted of a pre-treatment with either LPS (0.8 ng/kg) or saline PBO administered intravenously at time t = 0 min. After 30 min, subjects received oral MP (40 mg). The MP + LPS/PBO [^11^C]raclopride scan was then performed 60 min after MP administration (90 min after LPS/PBO pre-treatment) for 90 min. The timing of LPS administration was selected to achieve maximal levels of plasma cytokines during PET scanning as previously described (Petrulli et al., 2017; Sandiego et al., 2015). Subjects returned at least 1-week later for a second scan session that followed the same sequence (LPS/PBO pre-treatment, MP administration, MP + LPS/PBO scan) but with the opposite pre-treatment condition (LPS/PBO). The order of pre-treatment was randomized and double-blinded. Six out of 8 subjects had scan sessions 8 days apart. The average was 22 (SD:28) days apart. Seven out of the eight subjects included received their baseline scan at their first PET scan session. Six out of the eight subjects received MP + PBO for their first post-MP PET scan session. Scan cancellations sometimes alter the original randomization. Order effects were not observed. The dose of MP was determined using previous studies that reported a robust change in raclopride signal (~10–30 % change in dopamine) (Volkow et al., 2001; Volkow et al., 2002). The dose of LPS was selected based on previous studies that produced changes in brain activity (Eisenberger et al., 2010; Hannestad et al., 2012) and altered the dopamine response to MP (Petrulli et al., 2017).

#### 2.1.3. Imaging data acquisition

Subjects were permitted a light breakfast (without caffeine) prior to scanning. Subjects were not permitted to use anti-inflammatory medications for at least 3 days prior to each scan session. An intravenous catheter was placed for [^11^C]raclopride injection and periodic blood sampling. Blood pressure, heart rate, and temperature were monitored pre-, during, and post-scanning by medical staff to ensure subject well-being.

PET was performed with the High-Resolution Research Tomograph (HRRT; Siemens/CTI; FWHM = 2–3 mm). [^11^C]raclopride was synthesized using methods previously described (Gallezot et al., 2014). Before each PET scan, a 6-min transmission scan was acquired for attenuation correction. [^11^C]raclopride was administered as a bolus by a computer-controlled pump (Harvard Apparatus, Holliston, MA, USA) and collection of emission data lasted for 90 min. Motion correction was applied using a Polaris Vicra (Northern Digital Inc.) (Carson et al., 2003) head-mounted device. The system measures and corrects motion, event-by-event.

The administered radiotracer had a mean activity of 19 ± 0.61 mCi and mean mass of 2.3 ± 0.30 μg. Injected activity was compared between conditions (baseline, MP + PBO, MP + LPS) using a single factor analysis of variance (ANOVA). We compared the change in mass between each of the pre-treatment conditions and baseline (MP + PBO – Baseline, MP + LPS – Baseline) using a student’s paired *t*-test. To determine the effect of mass on the D_2_ receptor, we used the highest [^11^C]raclopride concentration (in mass units) in the reference region (cerebellum) and the affinity (K_D_) of raclopride for the D_2_ receptor (Hietala et al., 1999), to calculate the largest possible occupancy of the D_2_ receptor by unlabeled raclopride (See Supplemental Material for the equation).

#### 2.1.4. Image reconstruction

PET emission data were collected in 3D. Two-dimensional sinograms were created with Fourier rebinning. Data were binned into 27 total time frames of 6 × 0.5 min, 3 × 1 min, 2 × 2 min, 16 × 5 min. Data were corrected for attenuation, scatter, dead time, detector sensitivity, and randoms. Images were reconstructed with ordered subset expectation maximization (OSEM) using 2 iterations and 30 subsets (in-house software; IDL) at a voxel size of 1.22 mm × 1.22 mm × 1.23 mm and image volume of 256 × 256 × 207 voxels.

#### 2.1.5. Image harmonization

To harmonize the current smoker dataset (acquired with the HRRT scanner) with the previously collected dataset in healthy controls (acquired with the HR+ scanner), we applied a harmonization method to make the HRRT images resemble HR+ images (as described in (Hoye et al., 2022). The harmonization method was a two-step process: (1) down-sampling the pixel size of the HRRT images to match the HR+ images, (2) filtering the downsampled images with an isotropic gaussian filter with 4.5 mm FWHM. The harmonized HRRT (hHRRT) images had image dimensions of 152 × 152 × 105 and a voxel size of 2.06 mm × 2.06 mm × 2.43 mm.

#### 2.1.6. Image analysis

Each hHRRT PET image was co-registered to the respective subject’s MR scan and then non-linearly registered to an MR template (MNI2) in a common space (Bioimage Suite (Papademetris et al., 2006). ROIs were extracted using automated anatomical labeling (Tzourio-Mazoyer et al., 2002) (AAL) to generate regional time activity curves (for cerebellum, whole striatum, caudate, putamen, and ventral striatum). Time activity curves were fitted with the simplified reference tissue model (Lammertsma and Hume, 1996) using the cerebellum as a reference region to calculate non-displaceable binding potential (BP_ND_). Change in BP_ND_ (ΔBP_ND_) was calculated between baseline and post-MP for both LPS and PBO pre-treatment conditions. Percent change in BP_ND_ is an approach that has been used conventionally with several dopamine radiotracers including [^11^C]raclopride, [^18^F]fallypride, [^11^C]PHNO, [^11^C]FLB457 PET, to quantify respective endogenous dopamine (Martinez et al., 2003; Okita et al., 2016; Zakiniaeiz et al., 2019; Whitton et al., 2020).

#### 2.1.7. Dopamine elevation

To compare differences in MP-induced dopamine elevation, a repeated-measures ANOVA (rmANOVA) featuring condition (MP + LPS vs MP + PBO) as a within-subjects factor was performed on ΔBP_ND_ values from our *a priori* ROI, the whole striatum. Each exploratory ROI within the striatum (ventral striatum, caudate, and putamen) was examined with a separate but identical rmANOVA *post-hoc*. The whole striatum was chosen *a priori* based on the previously reported effects of MP-induced dopamine elevation by Petrulli et al. (Petrulli et al., 2017). The subregions were exploratory ROIs and were not corrected for multiple comparisons. Kolmogorov-Smirnov and Shapiro-Wilk tests of normality were conducted to confirm that our ΔBP_ND_ values were normally distributed as this is a critical assumption of the rmANOVA model.

#### 2.1.8. Blood and behavior analyses

Samples of venous blood (10 ml) were drawn at 30, 90, 150, 210 min post-MP administration to assess MP concentration and cortisol levels. An additional 10 ml of venous blood samples were drawn at 0, 30, 60, 90, 120, 180, and 210 min post LPS/PBO injection to measure concentrations of the cytokines: tumor necrosis factor alpha (TNFα), interleukin 6 (IL-6) and IL-8. Blood samples were centrifuged for 10 min and the plasma was stored at −80 °C. Plasma samples were assayed for MP (National Medical Services (NMS) Labs, Horsham, PA, USA), and cortisol and cytokines (Yale Center for Clinical Investigations (YCCI) Lab, New Haven, CT, USA).

To confirm that MP plasma levels were the same in both conditions, we compared MP plasma levels within subjects between MP + LPS and MP + PBO conditions at all timepoints using student’s paired *t*-tests. To check that LPS elevated cortisol and cytokine levels relative to PBO, we compared cytokine and cortisol levels at all timepoints between MP + LPS and MP + PBO conditions using student’s paired *t*-tests.

Subjects reported subjective ratings of endotoxin symptoms (headache, muscle pain, shivering, nausea, breathing difficulties, and fatigue) on a 0 to 4 scale at 0, 60, 90, 120, and 180 min post LPS/PBO injection and at the end of the scan day.

### 2.2. Study 2:

We combined the 8 smokers above with 8 healthy control subjects from the previously published study from our group (Petrulli et al., 2017). We examined this combined sample of 16 subjects to determine power and effect size in a heterogenous sample.

#### 2.2.1. Combining smoker and healthy control groups

Before combining smoker and healthy control groups, a number of data checks were performed (Table 1). Differences in age, sex, and scan order were assessed between the two groups using Chi-squared tests. Differences in bodyweight, LPS dose, LPS dose per bodyweight, MP dose per bodyweight, and subjective responses to endotoxin symptoms were assessed using student’s independent-samples *t*-tests. Cytokine and MP plasma levels were also compared between groups using independent-samples student’s *t*-tests at each time point. Cortisol plasma levels were not collected in healthy control subjects and thus, were not compared between groups. Injected activity and difference in injected mass between baseline and MP + LPS and baseline and MP + PBO were compared using student’s independent-samples *t*-tests.

#### 2.2.2. Dopamine elevation

To compare differences in MP-induced dopamine elevation in the combined sample, identical rmANOVAs featuring condition (MP + LPS vs MP + PBO) as a within-subjects factor was performed on whole striatum ΔBP_ND_ values, as well as each exploratory ROI (ventral striatum, caudate, and putamen) as described above.

To determine if smoking status affected the ΔBP_ND_, smoking status was added as a between-subjects variable in the rmANOVA model. The rmANOVA was the most appropriate statistical test due to the within-subjects design. Nonetheless, a two-way ANOVA was also used to examine the effect of smoking status. To evaluate whether having only one baseline scan in the Petrulli et al. (Petrulli et al., 2017) study versus two baseline scans would have changed any findings of significance, we arbitrarily chose the first acquired baseline scan to calculate ΔBP_ND_ between baseline and MP + LPS/PBO conditions and performed the same rmANOVA.

## 3. Results

### 3.1. Study 1:

#### 3.1.1. Dopamine elevation

**Fig. 2.**
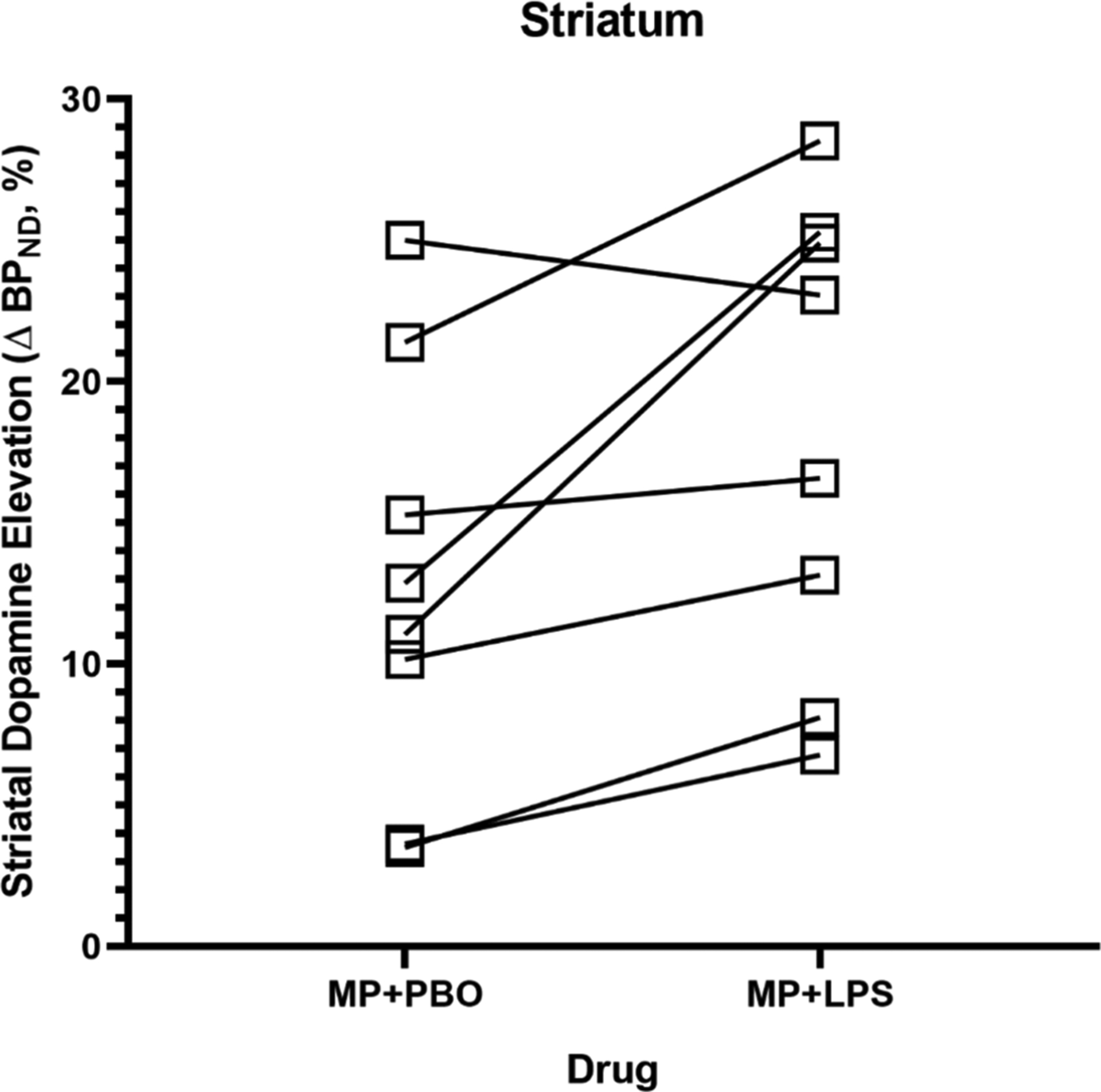
Smokers ΔBP_ND_ values comparison for MP + PBO and MP + LPS conditions. Smokers showed significantly higher ΔBP_ND_ values under the MP + LPS condition compared to the MP + PBO, *p <* 0.001.

The rmANOVA demonstrated a significant effect of condition (MP + LPS vs MP + PBO; *p* = 0.025, $\eta_{\text{p}}^{2}=0.54$) in the whole striatum whereby dopamine elevation was significantly higher under the MP + LPS (ΔBP_ND_ = 18 ± 2.9 %) condition compared to the MP + PBO (ΔBP_ND_ = 13 ± 2.7 %) condition (Fig. 2). In exploratory ROIs, there was a significant effect of condition for putamen, ventral striatum, and caudate (*p* = 0.013, *p* = 0.035, *p* = 0.005 respectively; uncorrected).

#### 3.1.2. Blood and behavioral analysis

Mean plasma MP levels were not different at any timepoints between MP + LPS and MP + PBO conditions (p > 0.35). Mean plasma cortisol levels were not different at 0, 30, and 60 min post-LPS injection. For all other timepoints, mean cortisol levels were significantly higher (or trending higher) for the MP + LPS compared to MP + PBO condition (*p <* 0.05 or *p <* 0.08); Figure S1). Mean cytokine levels were not different at 0 and 30 min (0.18 < *p* > 0.46) post-LPS injection between MP + LPS and MP + PBO conditions. For all later timepoints (60–210 min), mean cytokine levels were higher (although not always significantly) for the MP + LPS compared to MP + PBO condition (0.72 < p > 0.005; Table S2). Subjective responses of endotoxin symptoms were nonexistent to mild across conditions and groups (maximum mean rating for any symptom at any timepoint = 0.78).

#### 3.1.3. Injection parameters

Injected activity was not significantly different between conditions (baseline, MP + PBO, MP + LPS) scans (*p* = 0.22). The change in mass between baseline and drug scan was not significantly different between conditions (MP + PBO–Baseline, MP + LPS–Baseline) as characterized by a student’s *t*-test (*p* = 0.78). The largest calculated occupancy of the D_2_ receptor by raclopride (mass) was 6.6 %.

### 3.2. Study 2:

#### 3.2.1. Combining smoker and healthy control groups

Age, sex, scan order, bodyweight, LPS dose, LPS dose per bodyweight, MP dose per bodyweight, and subjective responses to endotoxin symptoms were not different between groups (p > 0.24; Table 1). Cytokines were similarly elevated post-LPS injection in both smoker and healthy control groups (Table S2). MP plasma levels were not different between groups at all timepoints (p > 0.33). Injected activity and difference in injected mass between baseline and MP + LPS and baseline and MP + PBO were not different between groups (p > 0.15). These data checks determined that the groups were well-matched and appropriate to be combined.

#### 3.2.2. Dopamine elevation

**Fig. 3.**
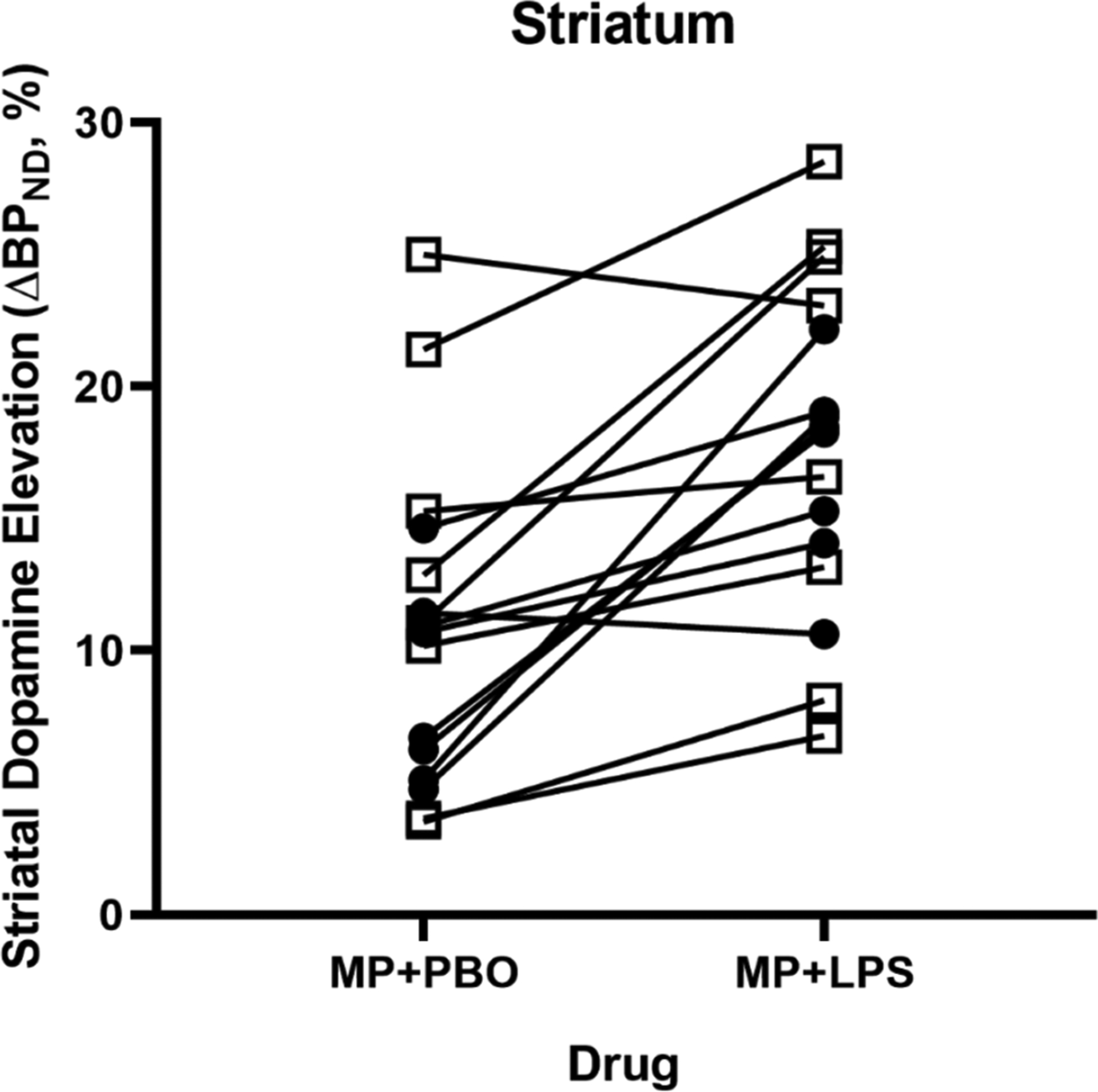
Smoker and healthy control (combined sample) ΔBP_ND_ values comparison for MP + PBO and MP + LPS conditions. Subjects showed significantly higher ΔBP_ND_ values under the MP + LPS condition compared to the MP + PBO, *p <* 0.001. Smokers are shown in open squares and healthy controls are shown in closed circles.

The rmANOVA demonstrated a significant effect of condition (MP + LPS vs MP + PBO; *p <* 0.001, $\eta_{\text{p}}^{2}=0.62$) in the whole striatum whereby dopamine elevation was significantly higher under the MP + LPS (ΔBP_ND_ = 18 ± 1.6 %) condition compared to the MP + PBO (ΔBP_ND_ = 11 ± 1.5 %) condition (Fig. 3) in the combined sample. In exploratory ROIs, there was a significant effect of condition for putamen (*p <* 0.001) and caudate (*p* = 0.003), but not ventral striatum (*p* = 0.111), uncorrected.

The rmANOVA with smoking status as a between-subjects variable demonstrated that the main effect of smoking status was not significant for whole striatum (*p* = 0.35) or for exploratory ROIs (*p >* 0.27) and that smoking status did not interact with the effect of condition for the whole striatum (*p* = 0.35) or for exploratory ROIs (*p >* 0.21). Using a two-way ANOVA does not change the significance of any reported findings (*p*’s > 0.32). Based on power analyses (Supplemental Material), our study is sufficiently powered to detect condition and group differences of interest, despite the small sample size. Using the first acquired baseline scan for the healthy control subjects (Petrulli et al., 2017) did not change any findings of significance compared to using both baseline scans.

## 4. Discussion

This is the first double-blind, randomized, placebo-controlled, cross-over study to examine MP + LPS-induced dopamine elevation in tobacco smokers. LPS administration produced an acute systemic inflammatory response and biological stress as evidenced by elevated cytokine and cortisol levels under the LPS condition compared to PBO, consistent with prior studies examining healthy control subjects from our group and others (Eisenberger et al., 2010; Petrulli et al., 2017; Hannestad et al., 2012; DellaGioia et al., 2013; Hannestad et al., 2011; Bach et al., 20162016). Subjective ratings of LPS symptoms were mild, and consistent with subjective ratings in our previous MP + LPS study (Petrulli et al., 2017). In Study 1, as hypothesized, MP-induced dopamine elevation was amplified by LPS in the striatum, replicating and translating our previous finding in 8 healthy control individuals (Petrulli et al., 2017) to tobacco smokers. In Study 2, we combined 8 smokers from the present study with the 8 healthy control individuals from the aforementioned study from our group. Groups were well-matched on demographics, injection parameters, and subjective responses to LPS. Smoking status did not interact with the effect of condition. In the combined cohort of 16 subjects, MP-induced dopamine elevation in the striatum was amplified in the presence of LPS, suggesting that acute systemic inflammation by LPS magnifies the dopamine response in both smokers and healthy controls.

Building on our prior work in healthy control individuals from our group (Petrulli et al., 2017), we replicated and translated our finding that dopamine elevation is amplified by an acute dose of LPS to an independent cohort of tobacco smokers. Our central finding links LPS-induced neuroimmune activation, a type of biological stress, to hyper-responsivity of the dopamine system in tobacco smokers. To our knowledge, this was the first study to assess the impact of an acute laboratory-based stressor on the reward system of tobacco smokers. The application of our LPS-based human laboratory model was successful in eliciting an acute systemic inflammatory stress response in tobacco smokers and may be applicable to other addicted samples, generally.

Although not studied here, we believe the distinction between chronic and acute inflammation is important. Petrulli et al. (Petrulli et al., 2017) found that an acute dose of LPS (one dose 1.5 h prior to scanning) in human subjects resulted in amplified striatal dopamine elevation relative to placebo (Petrulli et al., 2017). Felger et al. (Felger et al., 2013) found that chronic (4 week) administration of inflammatory cytokines in rhesus monkeys resulted in lower striatal dopamine release relative to placebo. However, that same study also showed that short-term (2 weeks) administration resulted in increased striatal dopamine release relative to placebo (Felger et al., 2013). In rodents, acute systemic immune activation with LPS produced increases in dopamine (Dunn, 1992). The exact biological distinction between acute and chronic inflammation is still unknown. It is possible that acute doses of inflammation may cause turnover of neuroendocrine peptides resulting in short-term higher dopamine release prior to long-term lower dopamine release (Felger, 2018).

Although our findings were similar between smokers and healthy controls, our study identified subtleties that might have implications for smoking reinforcement, consequences of drug use, and treatment. We observed a difference in the ventral striatum between smokers and healthy control individuals. Study 1 revealed that relative to PBO, LPS amplified MP-induced dopamine elevation in tobacco smokers in ventral striatum. Our previously published findings in healthy control individuals (Petrulli et al., 2017) did not detect differences between LPS and PBO conditions in the ventral striatum. Ventral striatum is a critical component of the reward-related circuitry and dopamine release in ventral striatum underlies the reinforcing properties of tobacco smoking. It is possible that LPS amplification of the dopamine signal in ventral striatum may explain why tobacco smokers continue to smoke or perhaps this may be a consequence of chronic smoking that may hinder responses to treatment. Because biological stress, such as immune activation, increases the likelihood of smoking relapse (McKee et al., 2011; Sinha, 2001), this finding suggests that ventral striatum may be an understudied leading edge in identifying how systemic inflammation and biological stress affect tobacco smokers differentially.

The present study was designed to assess the effects of systemic inflammation on dopamine in tobacco smokers and was not designed to directly assess neuroinflammation in smokers. The effects of smoking on neuroinflammation are not fully understood and may depend on abstinence time. Brody et al. (2017) showed that smokers have lower TSPO receptor availability than healthy controls as measured with [^11^C] DAA1106. A similar TSPO receptor availability study by Hillmer et al. (2020) found no difference in TSPO levels between smokers and healthy controls. In Brody et al. smokers were scanned in a satiated state and in Hillmer et al. smokers were abstinent for 2–14 h. Future studies should assess TSPO receptor availability in smokers pre-treated with LPS compared to placebo and the effect of smoking abstinence.

This study had some potential limitations that were addressed as well as several strengths. First, the comparison of smokers in Study 1 and healthy controls in Study 2 was limited by the use of two different PET scanners. However, we went to great lengths to harmonize the image resolution by scanning an independent set of subjects on both scanners (Hoye et al., 2022). This was essential to produce an appropriate comparison to our previous data. Other groups have taken similar steps to harmonize data acquired with different PET scanners (Joshi et al., 2009; van Velden et al., 2015). Another potential limitation was our small sample size of 8 smoking subjects. However, large effect sizes, high power, and our finding in the combined sample of 16 healthy control subjects gives us confidence. We demonstrated that our LPS model is capable of amplifying the dopamine response and serving as an immune activator and biological stressor in smoker and healthy control samples in a laboratory setting. It will be important for future work to examine how the magnitude of dopamine release might be related to smoking-related behavioral variables. Future studies should consider exploring the effect of LPS on other neurotransmitter systems (beyond dopamine). Future studies should adopt this LPS model to examine the interactions of the immune and dopamine systems in other neuropsychiatric populations. Future work should also study groups that have suffered chronic stress or immune activation through natural events and causes.

## 5. Conclusions

MP-induced dopamine elevation in the striatum was amplified by the administration of LPS to tobacco smokers and in the combined sample of smokers and healthy controls. The application of our LPS-based human laboratory model was successful in eliciting an acute systemic inflammatory response translating our previous findings in an independent sample of addicted individuals. The effect of inflammation on dopamine release could have important implications in drug reinforcement.

## Supplementary Material

Supplementary Material

## Figures and Tables

**Table 1:** Subject demographics. Smokers and healthy controls were well-matched on demographics, drug dosing, and scanning parameters.

	Study 1: Smokers (N = 8)	Study 2: Healthy Controls (N = 8)	Smokers v. Healthy Controls *p*-value
Sex (N female)	6	4	0.64
Age	33 ± 4.0	31 ± 3.9	0.79
Cigarettes/day	15 ± 2.9	–	–
Years smoked	16 ± 4.2	–	–
FTND	5.8 ± 0.8	–	–
Scan Order (N PBO first)	6	4	0.30
Bodyweight (kg)	75 ± 6.0	69 ± 4.7	0.42
LPS dose (ng)	60 ± 4.7	55 ± 3.7	0.40
LPS dose/bodyweight (ng/kg)	0.80 ± 0.0006	0.80 ± 0.003	0.25
MP/bodyweight (mg/kg)	0.56 ± 0.04	0.60 ± 0.05	0.48
Injected Activity (mCi)	19 ± 0.61	17.4 ± 0.61	0.15
ΔInjected Mass (PBO-Baseline; μg)	0.34 ± 0.88	0.20 ± 0.08	0.87
ΔInjected Mass (LPS-Baseline; μg)	0.05 ± 0.57	0.10 ± 0.09	0.94

ΔInjected Mass refers to the difference in the mass of the radiotracer injected between the conditions described. FTND = Fagerström Test for Nicotine Dependence. Mean ± SE shown.

## Data Availability

The data used in this study are not openly available at this time because the study is ongoing. Software and code are available upon request.
