# Supplementary material for: Systemic inflammation enhances stimulant-induced striatal dopamine elevation in tobacco smokers
Source: Brain Behav Immun. Author manuscript; Available in PMC 2023 Apr 12. (PMC10097458; doi:10.1016/j.bbi.2022.08.016)
Supplement: Supplementary Material [file NIHMS1888802-supplement-Supplementary_Material.docx]

**Supplemental Material**

1. Demographics with excluded subject (**Table S1**)
2. Sleep Deprivation
3. Occupancy Formula
4. Cortisol Levels over Time (**Figure S1**)
5. Cytokine Levels over Time (**Table S2**)
6. Power Analysis

1. **Demographics with excluded subject**

|  | **Study 1: Smokers (N=9)** | **Study 2: Healthy Controls (N=8)** | **Smokers v. Healthy Controls *p*-value** |
| --- | --- | --- | --- |
| Sex (N female) | 6 | 4 | 0.64 |
| Age | 32±3.6 | 31±3.9 | 0.34 |
| Cigarettes/day | 14±2.8 | - | - |
| Years smoked | 15±3.7 | - | - |
| FTND | 5.7±0.8 | - | - |
| Scan Order (N PBO first) | 7 | 4 | 0.34 |
| Bodyweight (kg) | 74±5.4 | 69±4.7 | 0.48 |
| LPS dose (ng) | 59±4.3 | 55±3.7 | 0.46 |
| LPS dose/bodyweight (ng/kg) | 0.80±0.0005 | 0.80±0.003 | 0.24 |
| MP/bodyweight (mg/kg) | 0.56±0.04 | 0.60±0.05 | 0.52 |
| Injected Activity (mCi) | 19±0.54 | 17.4±0.61 | 0.12 |
| ΔInjected Mass (PBO–Baseline; ug) | 0.17±0.79 | 0.20±0.08 | 0.98 |
| ΔInjected Mass (LPS–Baseline; ug) | 0.01±0.51 | 0.10±0.09 | 0.88 |

**Table 1: Subject demographics.** Smokers and healthy controls were well-matched on demographics, drug dosing, and scanning parameters. ΔInjected Mass refers to the difference in the mass of the radiotracer injected between the conditions described. FTND = Fagerström Test for Nicotine Dependence. Mean±SE shown.

1. **Sleep Deprivation**

One subject from the tobacco smoker group was excluded from this study due to possible disturbance of sleep prior to one of his scans. The subject was a shift worker. Volkow et al. (2012) found that in healthy subjects ventral striatal D_2_R availability (measured with [^11^C]raclopride PET) was significantly lower on days following sleep-disturbance compared to following well-rested nights. In other words, baseline scans acquired on days following sleep-disturbance are not comparable to those following well-rested nights. The excluded subject’s baseline and MP+LPS scans occurred on a day following a well-rested night and the MP+PBO scan occurred on a day following a night of disturbed night. The subject’s striatal MP+LPS ΔBP was -1.10 and MP+PBO ΔBP was 10.8. Given the Volkow et al. (2012) finding, we postulated that this subject's MP+PBO scan was invalid; thus, he was excluded.

1. **Occupancy Formula**

$Occupancy=\frac{[raclopride ]}{\left[ raclopride \right]+K_{D}}$ , where [raclopride] is the concentration of raclopride (nM) in the tissue and K_D_ is the equilibrium dissociation constant for raclopride at the D_2_ receptor (nM). We approximate the maximum occupancy by using the maximum molar concentration of raclopride in the cerebellum where the signal is assumed to be entirely free (and not bound) raclopride.

1. **Cortisol Levels over Time**


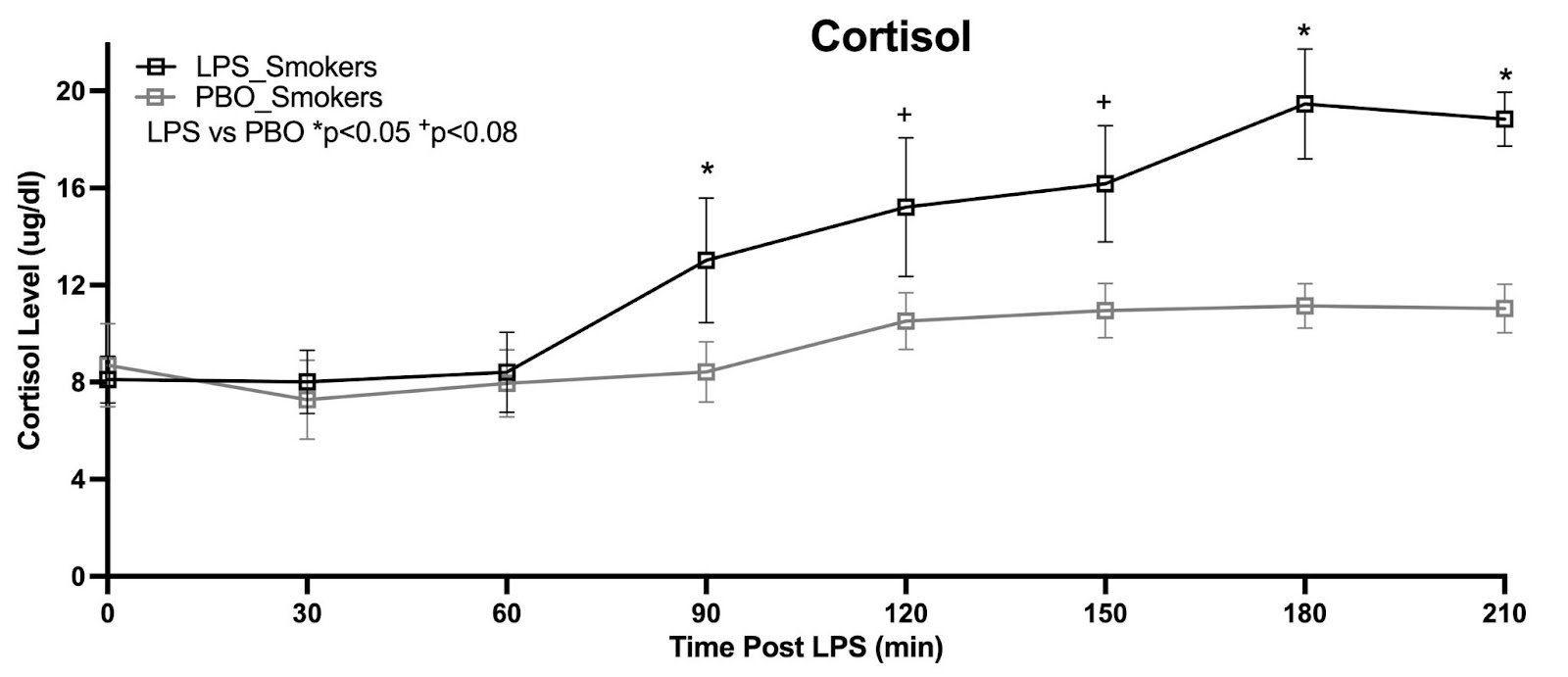


**Figure S1: Cortisol Levels Over Time.** Plasma cortisol levels (ug/dl) at 0, 30, 60, 90, 120, 180 and 210min post LPS injection. Mean and SE error bars shown.

1. **Cytokine Levels Over Time**

| **Cytokine (pg/ml)** | **Time (min)** | **Study 1: Smokers PBO** | **Study 1: Smokers LPS** | **Study 1: PBO vs. LPS *p*-value** | **Healthy Controls LPS** | **Smokers v. Healthy Controls LPS *p*-value** |
| --- | --- | --- | --- | --- | --- | --- |
| TNFɑ | 0 | 7.9±1.6 | 6.8±1.9 | 0.37 | 6.7±0.9 | 0.96 |
| TNFɑ | 30 | 8.2±2.2 | 10±2.9 | 0.39 | 6.5±1.2 | 0.30 |
| TNFɑ | 60 | 7.9±1.7 | 69±16 | **0.005** | 94±19 | 0.34 |
| TNFɑ | 90 | 11±4.3 | 75±19 | **0.008** | 134±15 | **0.03** |
| TNFɑ | 120 | 11±5.0 | 90±26 | **0.02** | 91±18 | 0.98 |
| TNFɑ | 180 | 7.7±1.6 | 58±16 | **0.009** | 71±14 | 0.57 |
| TNFɑ | 210 | 6.4±1.4 | 48±12 | **0.02** | 82±7.1 | **0.03** |
| IL-6 | 0 | 9.3±6.1 | 7.9±6.0 | 0.13 | 3.1±1.4 | 0.47 |
| IL-6 | 30 | 9.1±5.7 | 8.5±6.0 | 0.46 | 1.4±0.3 | 0.28 |
| IL-6 | 60 | 11±7.3 | 4.7±1.9 | 0.42 | 9.8±4.1 | 0.26 |
| IL-6 | 90 | 11±6.4 | 11±4.7 | 0.99 | 67±20 | **0.03** |
| IL-6 | 120 | 11±6.1 | 34±17 | 0.13 | 126±29 | **0.01** |
| IL-6 | 180 | 12±8.0 | 21±12 | 0.13 | 95±20 | **0.005** |
| IL-6 | 210 | 15±9.2 | 19±11 | 0.48 | 92±9.6 | **0.0001** |
| IL-8 | 0 | 31±17 | 29±15 | 0.18 | 5.0±2.2 | 0.17 |
| IL-8 | 30 | 29±15 | 28±15 | 0.25 | 3.1±0.6 | 0.13 |
| IL-8 | 60 | 33±18 | 19±11 | 0.39 | 5.7±1.0 | 0.27 |
| IL-8 | 90 | 32±16 | 39±16 | 0.72 | 35±8.2 | 0.84 |
| IL-8 | 120 | 31±15 | 109±44 | 0.06 | 123±34 | 0.81 |
| IL-8 | 180 | 35±19 | 65±36 | 0.17 | 134±44 | 0.24 |
| IL-8 | 210 | 39±20 | 62±26 | 0.10 | 154±28 | **0.03** |

**Table S2: Cytokine Levels Over Time.** Plasma cytokine (TNFɑ, IL-6, IL-8) levels (pg/ml) at 0, 30, 60, 90, 120, 180 and 210min post LPS/PBO injection. Mean±SE shown, uncorrected *p* values.

1. **Power Analysis**

Power analyses were conducted *post-hoc* for striatal ΔBP_ND_ values (α=0.05). Repeated measures ANOVAs were performed using the software, G*Power, Version 3.1.9.6. In the healthy control sample (effect size: η_p_^2^=0.67), 8 subjects provided 99% power to detect differences between conditions. In the smoker sample (η_p_^2^=0.54), 8 subjects provided 99% power to detect differences between conditions. In the whole sample (effect sizes – within: η_p_^2^=0.62; between: η_p_^2^=0.09; within-between interaction: η_p_^2^=0.08), 16 subjects provided ≥90% power to detect differences between conditions, between groups, and an interaction between conditions and groups. Based on these analyses, we can conclude that our study is sufficiently powered to detect condition and group differences of interest despite the small sample size. This is due in part to very large effect sizes (η_p_^2^≈0.6) between conditions in both samples.
